# Twenty Years of AIRE

**DOI:** 10.3389/fimmu.2018.00098

**Published:** 2018-02-12

**Authors:** Roberto Perniola

**Affiliations:** ^1^Department of Pediatrics, Neonatal Intensive Care, Vito Fazzi Regional Hospital, Lecce, Italy

**Keywords:** animal disease models, autoimmune polyendocrinopathies, immune tolerance, thymus gland, transcription factors, type-1 diabetes mellitus

## Abstract

About two decades ago, cloning of the autoimmune regulator (*AIRE*) gene materialized one of the most important actors on the scene of self-tolerance. Thymic transcription of genes encoding tissue-specific antigens (ts-ags) is activated by AIRE protein and embodies the essence of thymic self-representation. Pathogenic AIRE variants cause the autoimmune polyglandular syndrome type 1, which is a rare and complex disease that is gaining attention in research on autoimmunity. The animal models of disease, although not identically reproducing the human picture, supply fundamental information on mechanisms and extent of AIRE action: thanks to its multidomain structure, AIRE localizes to chromatin enclosing the target genes, binds to histones, and offers an anchorage to multimolecular complexes involved in initiation and post-initiation events of gene transcription. In addition, AIRE enhances mRNA diversity by favoring alternative mRNA splicing. Once synthesized, ts-ags are presented to, and cause deletion of the self-reactive thymocyte clones. However, AIRE function is not restricted to the activation of gene transcription. AIRE would control presentation and transfer of self-antigens for thymic cellular interplay: such mechanism is aimed at increasing the likelihood of engagement of the thymocytes that carry the corresponding T-cell receptors. Another fundamental role of AIRE in promoting self-tolerance is related to the development of thymocyte anergy, as thymic self-representation shapes at the same time the repertoire of regulatory T cells. Finally, AIRE seems to replicate its action in the secondary lymphoid organs, albeit the cell lineage detaining such property has not been fully characterized. Delineation of AIRE functions adds interesting data to the knowledge of the mechanisms of self-tolerance and introduces exciting perspectives of therapeutic interventions against the related diseases.

## Introduction

Surface receptors, enzymes, hormones, structural proteins, and other molecules act as self-antigens (self-ags) and are susceptible to autoimmune targeting in adverse circumstances. In a significant number of cases, these substances are restricted to specific tissues and for this reason are named tissue-specific antigens (ts-ags). The notion that ts-ag-encoding genes are transcribed and translated into their respective proteins within the thymus, the so-called promiscuous gene expression (PGE), dates back to the eighties, when neurohypophyseal hormones, insulin-like growth factors, and other ts-ags were found in the human and animal gland (1–4).

Later, a quantitative correlation between PGE and negative selection was established: in 1997, two research groups assayed human insulin gene (*INS*) expression in thymi of aborted fetuses and children dead at various ages. The researchers found that the allele classes of the variable number of tandem repeats (*VNTRs*) upstream of *INS* promoter, the so-called type-1 diabetes (T1D) susceptibility locus 2, affected *INS* transcription, and suggested that higher amounts of thymic insulin could promote a more effective purge of the related self-reactive thymocyte clone (5–7). Similar studies supplied valuable data on thymic PGE and led to identify several markers of autoimmunity, but did not realize the extent of the phenomenon (8–10).

In 1998, Sospedra et al. stated that the human thymus contains self-ags belonging to three classes: those synthesized in peripheral tissues and circulating at high, moderate, or low concentration; those synthesized in peripheral tissues and ordinarily undetectable in the circulation; finally, secluded self-ags, such as the retinal-S antigen and the myelin basic protein (11). Noticeably, PGE amount showed marked inter-individual variability, as confirmed by later studies (12, 13).

In 2001, Derbinski et al. assayed the expression of a large set of ts-ag-encoding genes in murine thymic stromal cells: cortical and medullary thymic epithelial cells (cTECs and mTECs, respectively), dendritic cells (DCs), and macrophages. All gene transcripts were found in mTECs, and around 50% of them were restricted to this cell sublineage (14). Detection of mRNAs from five selected genes was first obtained in 15-embryonic-day (15E) embryos and persisted into late adulthood. PGE was enhanced in UEA1^hi^ mTECs (UEA1 stays for *Ulex europaeus* agglutinin 1). UEA1 labeling, in turn, was related to the co-stimulatory cluster of differentiation CD80, and, to a lesser degree, to class-II major histocompatibility complex (MHCII) antigens. Importantly, the expression of the autoimmune regulator (*Aire*) gene, which encodes the homonymous transcription factor, exhibited close distribution and timing (14), so that the study prompted the scientific community to inquire into the role of Aire in thymic self-representation and tolerance.

The present review is devoted to the fundamental aspects of Aire action and adverse consequences caused by its deficiency. Unless referring to the human counterparts (*AIRE* gene and AIRE protein), author will cite ordinarily murine gene (*Aire*) and protein (Aire), as the main body of scientific studies on this topic has been carried out on the animal models of disease. With regard to PGE, which is only in part dependent on Aire, author refers the kind readers to excellent reviews that delineate its extent and principles (15–17).

## Ontogenesis of TECs

### Generation of Mature TECs

In the murine thymus, *Aire* mRNA and Aire are traceable since 14E–15E (14, 18–20). Interestingly, in one of these studies the authors were able to detect *Aire* transcripts on a first-strand cDNA panel from 11E embryos (19). In this sense, a Chinese research group found that *Aire* is expressed in undifferentiated embryonic stem cells (ESCs), where it is co-stained with the stage-specific embryonic antigen 1, and that such expression attenuates upon ESC differentiation (21, 22). In ESCs, Aire associates with the spindle apparatus and plays a critical role in mitotic events (23). Hidaka et al. reported similar findings in embryoid bodies (24).

**Figure 1:**
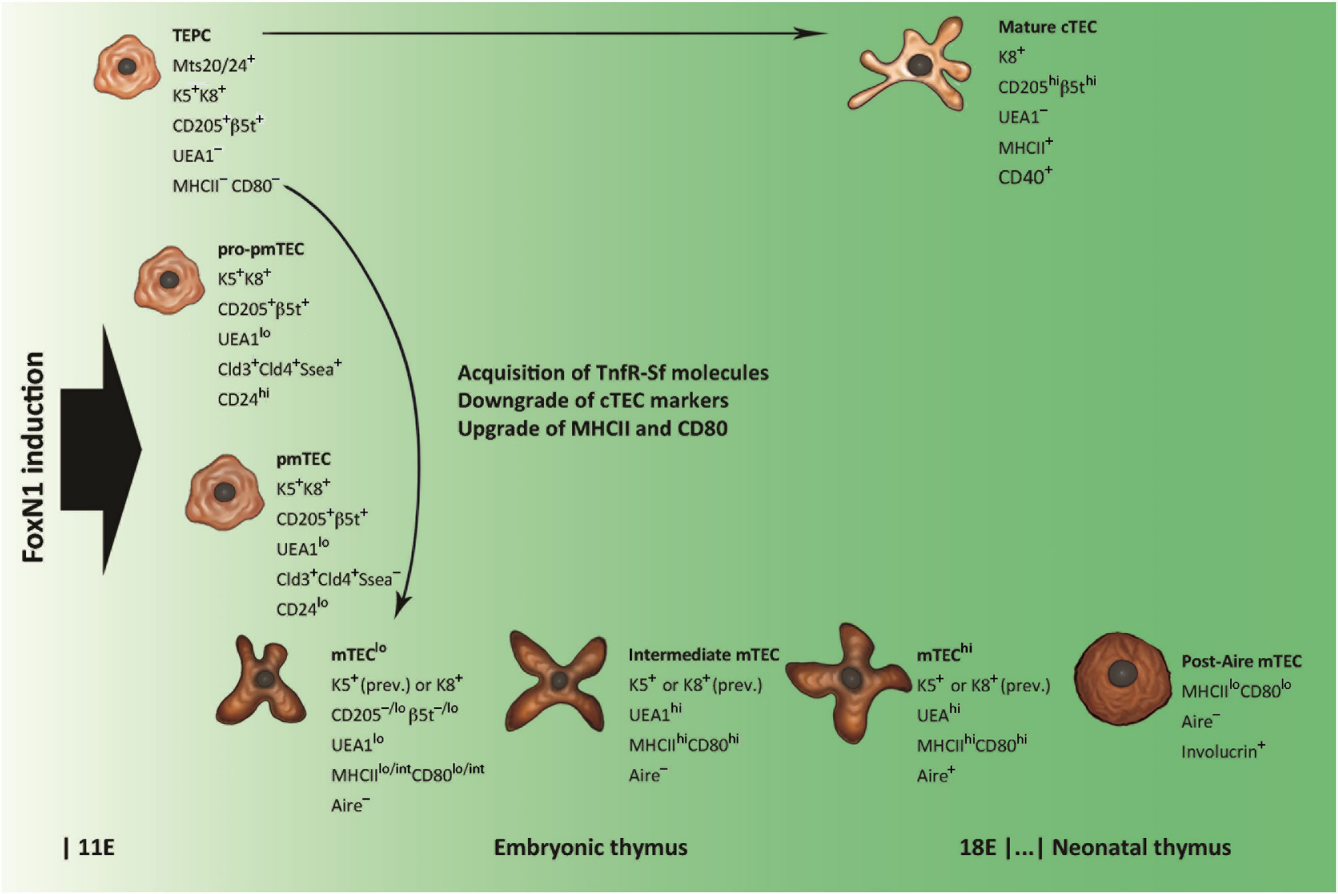
Schematic representation of thymic epithelial cell (TEC) differentiation. Thymic epithelial progenitor cell (TEPC) is tagged by mouse thymic stroma antibodies 20/24 (Mts 20/24), synthesizes intracellular keratins (Ks) 5 and 8 (K5 and K8, respectively), and exhibits surface markers associated with mature cortical TEC (cTEC), such as the cluster of differentiation CD205 and the thymoproteasome subunit β5t. Commitment to medullary TEC (mTEC) sublineage is restricted to claudine (Cld)-exposing elements, which, through intermediate stages of mTEC pro-precursor and precursor (pro-pmTEC and pmTEC, respectively), generate the immature mTEC (mTEC^lo^). mTEC^lo^ differentiation into mature mTEC (mTEC^hi^) is accompanied by enhancement of *Ulex europaeus* agglutinin 1 (UEA1) labeling and further upgrading of class-II major histocompatibility complex (MHCII) antigens and CD80. Lymphostromal interaction (thymic “crosstalk”) drives the emergence of pro-pmTECs by induction of molecules of the tumor necrosis factor-receptor super-family (TnfR-Sf), such as the lymphotoxin-β receptor (LtβR) and the receptor activator of nuclear factor Nf-κB (Rank). The transition from pro-pmTECs to pmTECs is characterized by loss of the stage-specific embryonic antigen 1 (Ssea) and results in a Rank^hi^ condition. Loss of *Aire* expression and acquisition of keratinocyte markers typify a subset of post-Aire mTECs that emerge in the postnatal thymus.

Many efforts have been produced to identify the thymic epithelial progenitor cells (TEPCs) from which Aire^+^ mTECs descend. Transplantation of endodermal cells of the third pharyngeal pouch from avian inter-species chimeras (25) and ectodermal-cell tracking in murine embryos (26) show that both cTECs and mTECs come from the endoderm, so that it is widely accepted that TEPCs are bipotent (27–31). In the simplest model of cTEC/mTEC commitment, TEPCs give rise simultaneously to sublineage-restricted elements. However, various research groups, on the basis of cTEC differentiation stages (32), have demonstrated that Aire^+^ mTECs derive from TEPCs exposing cTEC-associated markers, such as CD205, the thymoproteasome subunit β5t and the atypical CC-chemokines receptor (CCR)L1, and that such lineage persists in the postnatal thymus (33–36). Also interleukin (Il)7, which is required for T-cell development, is released by cTECs, and Il7^hi^ cTECs can generate CD80^+^ mTECs through Il7^–^CD80^lo^ elements (37). From this perspective, it has been possible to elaborate a model of cTEC/mTEC commitment in which mTEC sublineage diverges from a defaulted program of cTEC differentiation (38), as shown in Figure 1. Interestingly, in early organogenesis, the tight-junction claudins 3 and 4 mark the future Aire^+^ mTECs at the apex of the primordial endodermal layer (39). In the last few years, the researchers have focused their attention on TEPC characterization in the thymus of adult (at least 4-week-old) mice, applying different experimental settings and marker panels (40–45). Once again, markers of predetermined commitment to Aire^+^ mTECs have been identified (46, 47).

Finally, immature cTECs and mTECs deal with the differentiation program leading to full maturity. All TECs expose the epithelial-cell adhesion molecule (EpCAM), but, while mature cTECs have a rather homogeneous phenotype, two distinct mTEC subsets exist: UEA1^hi^ and UEA1^lo^ mTECs, also called mTECs^hi^ and mTECs^lo^, respectively (14, 32, 48–56). Distribution of keratins (Ks) into these subsets is not selective (48, 49, 55, 56); conversely, MHCII antigens and CD80 associate preferentially with the former (14, 50, 51, 54, 56). The expression of *Aire* and most ts-ag-encoding genes, in turn, is restricted to the mature, MHCII^hi^ or CD80^hi^, mTECs (14, 53, 56). These subsets represent about the same elements, which derive from their immature MHCII^lo^CD80^lo^ precursors (57–59). Proliferation markers and the pattern of regeneration after pharmacological ablation indicate that a stock of MHCII^hi^CD80^hi^Aire^−^ mTECs at an intermediate stage of differentiation exists (58–60), while predisposition to apoptosis suggests that Aire typifies terminally differentiated mTECs (57–61). Not in opposition to this evidence, later observations delineate a post-Aire mTEC stage, characterized by loss of *Aire* expression, suppression of PGE, reversion to MHCII^lo^CD80^lo^ condition, and synthesis of keratinocyte proteins, such as desmogleins and involucrin, a soluble precursor of the envelope of the epidermal stratum corneum (62–64).

### Transcriptional Regulation and Thymic “Crosstalk”

Although not fully known, there is a strict regulation of TEC ontogenesis. The thymic compartmentalization requires the transcription factor forkhead-box (Fox)N1, which is encoded at the “nude” locus: although referred to as athymic, the nude mice display an organ rudiment that includes TECs at an early stage of differentiation and is devoid of lymphoid progenitors (65). More recently, Nowell et al. have demonstrated that FoxN1, although dispensable for sublineage commitment, drives cTECs and mTECs along the program of differentiation (66). In the murine thymus, loss or downregulation of *FoxN1* expression subverts the organ morphology mimicking a precocious senescence (67, 68), while *FoxN1* upregulation reactivates TEPCs and reverses organ aging (69–71). These observations suggest that the thymic microenvironment reacts to FoxN1 in a dosage-sensitive manner and that *FoxN1* expression is regulated in accordance with age (72).

While cTEC differentiation is induced by thymocytes at an early stage of maturation, mTEC differentiation is dependent on their full maturation and relocation. This lymphostromal interaction, the so-called thymic “crosstalk,” is achieved through two pathways enabling the nuclear factors Nf-κB (73–75). Both pathways are triggered by intercellular signals between the tumor necrosis factor (Tnf) and tumor necrosis factor-receptor (TnfR) super-families (Tnf-Sf and TnfR-Sf, respectively). TnfR-Sf members exposed on mTEC surface are the lymphotoxin-β receptor (LtβR), the receptor activator of nuclear factor Nf-κB (Rank), and CD40. LtβR and CD40 are also exposed on cTEC surface (32). There is a bulk of experimental data from the studies on the role and consequences of loss and gain of function of these molecules in the embryonic and postnatal/adult thymus (76–114). The cited studies are those that, on a targeted basis, have evaluated the impact of these changes on the generation and differentiation of Aire^+^ mTECs.

Unsurprisingly, differences in murine strains employed, experimental settings, and timing of observation have produced contrasting results in a number of cases. However, recent studies have set out some basic principles, highlighting that LtβR and Rank cooperate in the embryonic thymus to switch TEPCs to mTEC sublineage, while in the following step mTEC precursors become Rank^hi^ (92, 95, 106). The release of the respective ligands is provided by T-cell subsets, such as lymphoid-tissue inducer cells and dendritic epidermal T cells, generated prior to the conventional αβ-thymocytes (98, 100, 102). Post-Aire mTEC differentiation and crosstalk of the postnatal thymus require inputs different from those acting in the embryonic period (91, 100, 101, 103). Presumably, thymic B cells and DCs participate in these processes (115, 116), while, if crosstalk is suppressed, coarse medullary cysts form, which are circumscribed by polarized mTECs (117). A careful dissection of the matter goes beyond the author scope, but essential aspects are reported in Table S1 in Supplementary Material. In addition, author refers the kind readers to excellent reviews that have thoroughly analyzed crosstalk dynamics, and the role and essentiality of each molecule involved (118–121).

Several other factors may exert inducing or inhibiting influence over mTEC development: of particular importance are the fibroblast growth factors (Fgfs), mainly Fgf7 (or keratinocyte growth factor), which is required for TEC differentiation in thymic organogenesis and regeneration (122). In murine models of graft-versus-host disease, administration of Fgf7 has proven to be decisive in the enrichment and maintenance of Aire^+^ mTECs able to promote T-cell reconstitution and avoid self-tolerance breaking (123–127).

Interestingly, mTEC differentiation may be reproduced *in vitro* by three-dimensional organotypic co-cultures engineered for dermal equivalent and based on the close relationship between skin and thymic stroma (128).

## AIRE Gene and the Related Protein

Human AIRE is encoded by a gene located in the region 22q.3 of chromosome 21 (129, 130). Pathogenic *AIRE* variants cause the autoimmune polyglandular syndrome type 1 (APS1), characterized by chronic surface candidiasis and various autoimmune diseases involving mainly the endocrine glands (131, 132).

Murine *Aire* maps to chromosome 10 in a region syntenic to human 21q22 (18–20). Similarly to the human gene, *Aire* expression is restricted to a few cells of the thymic medulla, represented by a significant percentage of mTECs^hi^, and, to a lesser degree, by DCs (76, 133). Presumably, Aire is synthesized and acts also in the secondary lymphoid organs, while, as reviewed elsewhere, *Aire* expression in other systems and cell lineages is uncertain and of doubtful meaning (134, 135).

### Biophysical and Biochemical Properties

**Figure 2:**
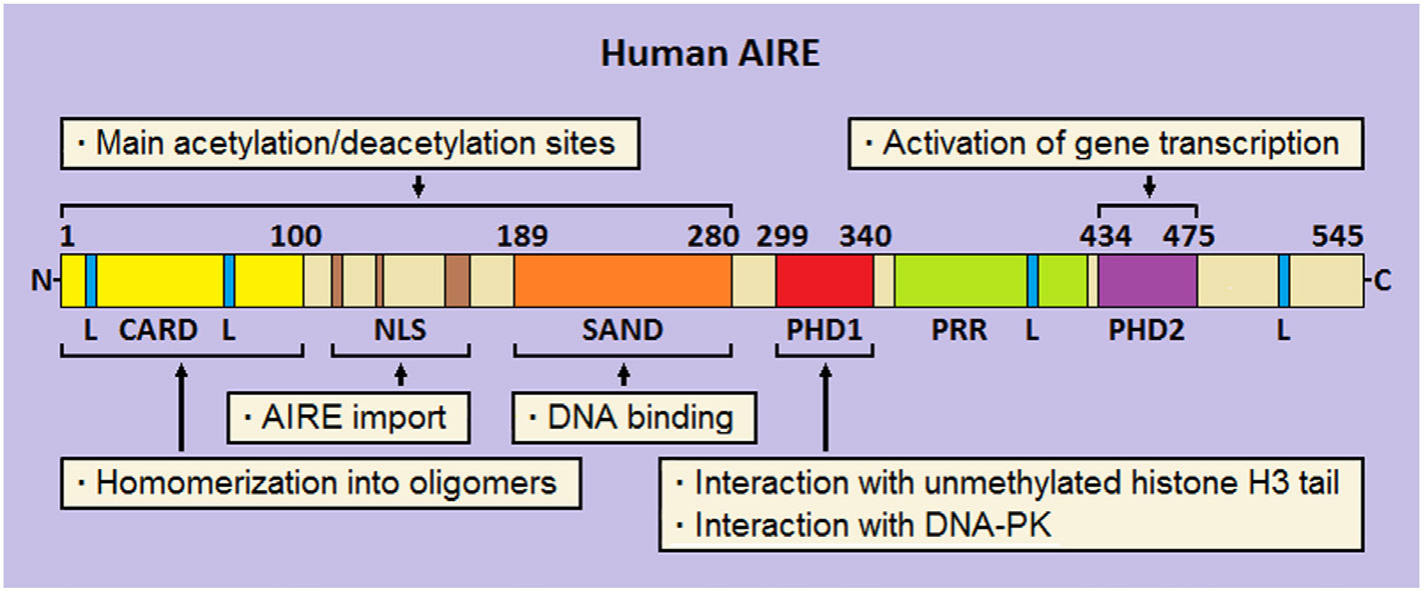
Schematic representation of human autoimmune regulator (AIRE). At the N-terminus, the caspase-activation and recruitment domain (CARD) and nuclear localization signal (NLS) are flanked by the SAND domain. Moving to the C-terminus, two plant-homeodomains (PHD1 and PHD2, respectively) fingers are separated by a proline-rich region (PRR). Four LxxLL (L stays for leucine) motifs are enclosed in the amino-acid chain. Preeminent domain-related properties are reported.

Analysis of its multidomain structure reveals that human AIRE belongs to the group of proteins able to bind to chromatin and regulate the process of gene transcription (136, 137). Starting from the N-terminus, AIRE comprises (Figure 2) a caspase-activation and recruitment domain (CARD), a nuclear localization signal (NLS), a SAND domain, and two plant-homeodomain (PHD) fingers (138). At subcellular level, AIRE localizes into small speckles uniformly distributed in the nucleoplasm and resembling the promyelocytic-leukemia nuclear bodies (NBs). In addition, it is visualized in the cytoplasm of a variable number of cells, where it forms a scaffold-like meshwork reminiscent of the intermediate filaments or microtubules (139–141). As observed in cultures of human mTECs and *AIRE*-transfected cell lines, AIRE has a subcellular organization following spatio-temporal cycles, and associates with the nuclear matrix (142, 143).

Homomerization into oligomers (dimers and tetramers) is an important biophysical property of AIRE, which allows binding to specific oligonucleotide motifs (144, 145). Suggestively, construction of a library of thymic consensus sequences highlighted that the promoters of several genes, among which those encoding ts-ags targeted by autoimmunity in *Aire*-deficient (*Aire*^−/−^) mice, enclose such motifs, albeit this mechanism represents a non-specific way of action of the protein (146).

Ability to homomerize is attributed to the AIRE N-terminus, already named homogeneously staining region (aa 1–100) by analogy with the speckled-protein SP100 (147). Two research groups demonstrated that pathogenic AIRE variants and deletion constructs involving this domain prevent oligomer formation and are unable to activate gene transcription (148, 149). Later, Ferguson et al. individuated in AIRE N-terminus a CARD (150), which is typical of pro-apoptotic proteins (151). Beside CARD, a bi- or tri-partite NLS guarantees AIRE shuttle into and out of the nucleus (152, 153).

In the middle of the amino-acid chain, the SAND domain (aa 180–280) encloses a basic amino-acid module that mediates AIRE binding to the phosphate groups of DNA (154), albeit SAND actual role is probably that of offering an anchorage to heterologous proteins (155). Importantly, CARD, NLS, and SAND domain hold most AIRE lysine residues, which are sites of acetylation (Ac) (145, 152, 153): this is a key point for proper protein localization and participation in multimolecular complexes.

At the C-terminus, AIRE is completed by two PHD fingers, named PHD1 (aa 299–340) and PHD2 (aa 434–475), which are separated by a proline-rich region. PHD fingers are cysteine-rich domains characterized by a four-cysteine, one-histidine, three-cysteine motif, which coordinates two zinc ions (156). In general, PHDs “read” the chromatin marks, mainly the degree of methylation at the tail of histone H3: importantly, AIRE PHD1 belongs to the PHD subfamily that recognizes unmethylation of H3 tail as a distinct epigenetic mark (157–159). At molecular level, opposite charges on the reciprocal surfaces facilitate the electrostatic interaction between PHD1 and H3 (160), while the methylation of some H3 amino-acid residues, mainly Arg2 and Lys4, dissociates them (161, 162). Despite a structural resemblance with PHD1, PHD2 displays a positively charged surface that makes it unsuitable to interact with histones (160). Nonetheless, its structural integrity is crucial for the activation of gene transcription, as confirmed by inherent AIRE variants (163) and deletion of the murine homolog (164). Actually, even the thirty amino acids positioned at the end of AIRE C-terminus act as an autonomous domain (165).

Finally, it should be remembered that AIRE encloses four LxxLL (L stays for leucine) motifs typical of proteins that bind to nuclear receptors and affect, as either co-activators or co-repressors, the transcriptional events (166). Interestingly, the fourth LxxLL motif lies in the C-terminus and is critical for AIRE properties (165).

### Molecular Mechanisms of Action

**Figure 3:**
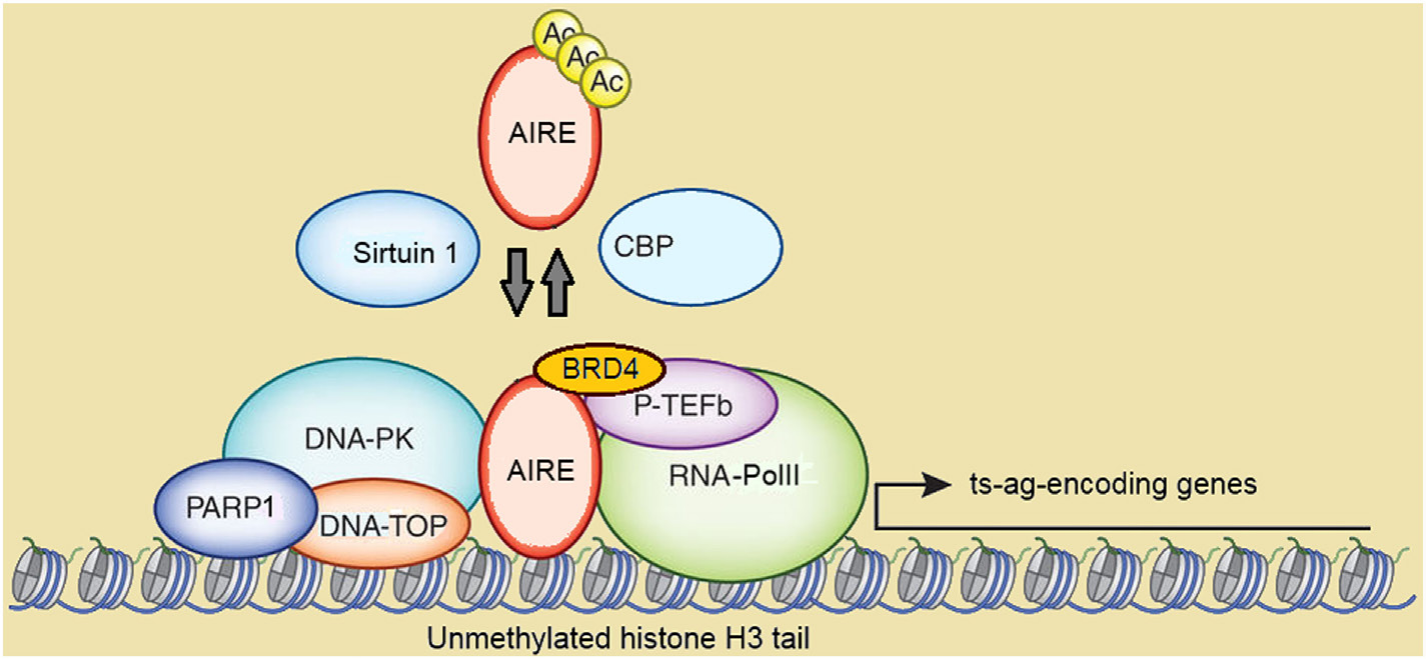
Schematic representation of autoimmune regulator (AIRE)-containing multimolecular complexes involved in initiation and post-initiation events of gene transcription. Abbreviations: Ac, acetylation; CBP, CREB-binding protein; DNA-PK, DNA-activated protein kinase; DNA-TOP, DNA-topoisomerase; PARP1, poly-(ADP-ribose) polymerase 1; BRD4, bromodomain-containing domain 4; P-TEFb, positive transcription elongation factor b; RNA-PolII, RNA-polymerase II; ts-ag, tissue-specific antigen. Reprinted (with changes) with the permission from Macmillan Publishers Ltd.: Peterson (171). Copyright 2015.

It is now clear that Aire does not act as a conventional transcription factor by binding to consensus sequences of the target gene promoters. Rather, the protein seems to participate in coordinated events performed by multimolecular complexes (Figure 3). Several studies have been produced to elucidate Aire’s partnerships and their functional relevance. An acceleration in this field has come from the study of Abramson et al., who used AIRE-targeted co-immunoprecipitation, mass spectrometry, and RNAi-mediated mRNA knockdown to identify the pool of associated proteins (167).

CREB-binding protein (CBP), which localizes in the NBs and is a co-activator of several transcription factors, was the first AIRE partner to be identified (148, 168). Following studies suggested that Ac by CBP stabilizes the subcellular distribution of AIRE, albeit data on targeted lysine residues and functional consequences were conflicting (169, 170). In a more recent study on murine mTECs, mapping of Aire lysine residues acetylated by CBP has been validated by bioinformatics-based candidate prediction. In this context, it has been highlighted that the group-III histone-deacetylase Sirtuin 1 preserves Aire-dependent PGE by deacetylation of such residues (171, 172).

Positive transcription elongation factor b (P-TEFb) and DNA-activated protein kinase (DNA-PK) are other AIRE partners (173–175). DNA-PK phosphorylates AIRE, at least *in vitro*, at Thr68 and Ser156 (174). Above all, DNA-PK belongs, together with other molecules co-immunoprecipitating with AIRE, to the multimolecular complex involved in DNA break and repair by non-homologous end joining (175). Among these molecules, a strong AIRE partner, as evidenced in proteomic assays, is the DNA-topoisomerase (DNA-TOP)IIα (167). DNA-TOPs are isomerase enzymes that operate on DNA topology and remove positive and negative DNA supercoils by generating transient DNA breaks: this causes local chromatin relaxation and facilitates the initiation and post-initiation events of gene transcription (176). DNA-TOPIIα performs double-stranded DNA breaks and attracts DNA-PK and poly-(ADP-ribose) polymerase 1 (PARP1). Recently, Bansal et al. have demonstrated that murine Aire and the above partners localize to long stretches of chromatin known as super-enhancers, which serve as depots of cell-specific multimolecular complexes involved in transcriptional events, and enclose the transcription start sites of most Aire-dependent genes. In the same study, the authors have indicated DNA-TopI, which introduces single-stranded DNA breaks, as a preeminent Aire partner upstream of DNA-TopIIα and DNA-TopIIβ (177). In another recent study, Guha et al. have clarified the details of the interaction between AIRE and DNA-TOPs: AIRE would exert a camptothecin- and etoposide-like function able to inhibit type-I and type-II DNA-TOP re-ligation activity. This is followed by chromatin changes attributable to DNA-PK and PARP1, and activates the transcription of low-expressed genes (178). Recently, a clinical picture resembling APS1 has been reported in two patients with pathogenic variants of the gene encoding the DNA-PK catalytic subunit. Unsurprisingly, PGE was impaired in patients’ fibroblasts transfected with *AIRE* (179).

Also the homeodomain-interacting protein kinase 2 (HIPK2), another serine-threonine protein kinase localized in the NBs, phosphorylates AIRE (and CBP) and exerts a repressive influence over the related properties. Interestingly, *Hipk2*-deficient (*Hipk2*^−/−^) mice undergo a PGE downgrade that mostly involves Aire-independent genes expressed in mTECs^lo^, suggesting that Hipk2 operates on hypothetical transcription factors other than Aire (180).

The interaction with P-TEFb seals AIRE participation in the post-initiation events of gene transcription (173). In eukaryotic cells, gene transcription is abortive if P-Tefb does not enable elongation and pre-mRNA splicing into mature mRNA by phosphorylation and release of stalled RNA-polymerase II. As observed in human and murine cell lines, AIRE recruits P-TEFb at the transcription start sites of the target genes and enables the above sequence (181, 182). Moreover, Yoshida et al. found that the bromodomain-containing protein 4 (Brd4) forms a bridge between murine Aire and P-Tefb, and that balanced phosphorylation and Ac of Aire CARD are necessary to keep such interaction (183). Finally, interaction with the human heterogeneous nuclear ribonucleoprotein L suggests that AIRE enhances mRNA diversity by favoring alternative mRNA splicing (184), as confirmed in murine mTECs (185, 186).

Although the studies so far examined have provided a formidable contribution to the knowledge of the molecular mechanisms of Aire action, how the protein recognizes the target genes remains to be fully explained. PHD1 disruption abrogates the transcription of a part of human AIRE-dependent genes (159), while a histone H3-specific demethylase does not enlarge their number (187), so that the hypothesis that promoters of AIRE-dependent and AIRE-independent genes merely differ in chromatin marks is unsatisfying (188). A complementary mechanism may be the interaction between AIRE and the complex formed by activating-transcription-factor-7-interacting protein (ATF7IP) and methyl-CpG-binding-domain protein 1 (MBD1) (189). ATF7IP can act as either co-activator or co-repressor of gene transcription depending upon its partners, while MBD1 belongs to a family of nuclear proteins able to bind to methylated CpG dinucleotides, which characterize the promoter region of silent or low-expressed genes. Thus, coopting such repressive complex would recruit AIRE to the target genes, but the details of the interaction need further explanation. In another study, murine chromatin enclosing Aire-dependent genes exhibited marks of polycomb silencing, such as histone H3 hypomethylation at Lys4 and trimethylation at Lys27. Although Aire partnership with chromodomain-helicase-DNA members, which bind to these amino-acid residues, is controversial (163, 167), it has been suggested that such putative interaction would drive Aire to the target genes and activate gene transcription by overriding a repressive chromatin state (190).

### Interaction with miRNAs

In the last few years, some research groups have put forward the hypothesis that Aire would be involved in post-transcriptional gene control by interaction with miRNAs, small (21–25 nucleotides in length) double-stranded non-protein-encoding RNAs, which join in silencing complexes able to cause translational block and mRNA degradation (191). TEC-restricted deletion of murine genes encoding molecules that participate in miRNA pathway makes the thymic environment unable to sustain thymocytes maturation and reach a proper PGE, with more or less obvious Aire dysregulation (192, 193). Observation of miRNA pattern changes throughout mTEC differentiation has led to identify a miRNA subset that affects specifically *Aire* mRNA translation (194–196). Conversely, Aire itself seems to condition amount and composition of miRNAs by modulating their transcription (197). Moreover, Aire would induce in genes involved in PGE a sort of refractoriness to the interaction with miRNAs, while in Aire deficiency a large number of miRNAs would achieve the target (198–200).

## Mechanisms of Action in Central Tolerance

**Figure 4:**
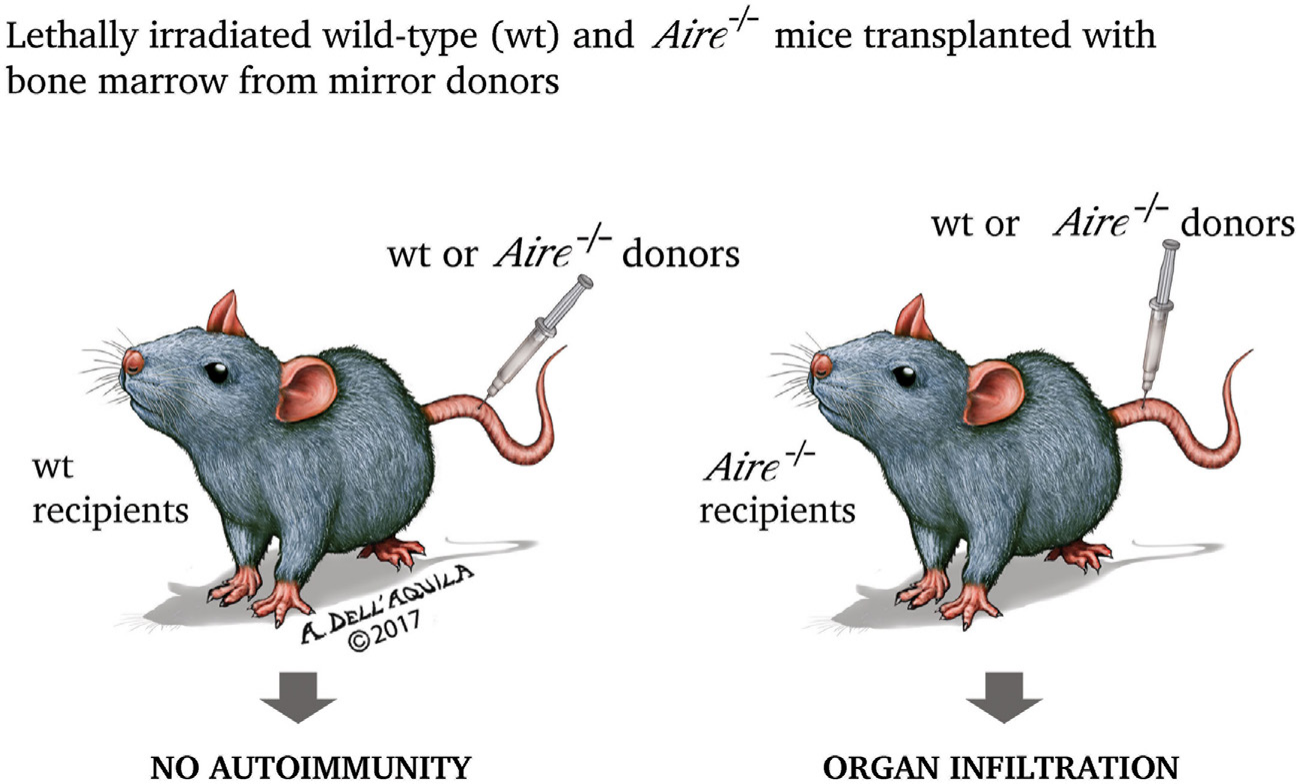
In the experiment by Anderson et al. (202), lethally irradiated (thus retaining only the radio-resistant stromal cells) wild-type and *Aire*^−/−^ mice were transplanted with bone marrow from mirror donors. Only *Aire*^−/−^ recipients, independently from the donor condition, exhibited organ damage, underlining that the property of preventing the autoimmune process is inherent to wild-type (*Aire*-sufficient) thymic stroma.

Once dissected the molecular mechanisms of Aire action, it is now appropriate to analyze in an orderly fashion the biological effects of such events. As expected, most information comes from studies on the animal models of disease. First *Aire*^−/−^ mice were engineered in 2002: the animals exhibited lymphocyte infiltration invading or surrounding specific structures of various organs (for example, the portal spaces of the liver, or the gastric parietal cells, or the outer layer of the retina), paralleled by circulating antibodies to self-ags with a similar, although not exactly corresponding, pattern (201, 202). When bone marrow from either wild-type (*Aire*-sufficient, *Aire*^+/+^) or *Aire*^−/−^ mice was transplanted into two mirror groups of lethally irradiated mice, organ infiltration and humoral autoimmunity were found only in *Aire*^−/−^ recipients, independently from the donor condition (Figure 4). Obviously, PGE was impaired in *Aire*^−/−^ thymi (202).

These principles were applied in a following study. Mice carrying T-cell receptor (TCR) loci immunized to the hen egg lysozyme were crossed with mice in which thymic expression of the related transgene was driven by the rat *Ins* promoter. The comparison between *Aire*^+/+^ and *Aire*^−/−^ double-transgenic mice revealed that the former had a small number of TCR-specific thymocytes, which exhibited anergy markers, while failure of negative selection in *Aire*^−/−^ mice caused spreading of the self-reactive T cells (203).

In the following subheadings, author will address the various modalities of Aire intervention in central tolerance: activation of PGE, presentation and transfer of self-ags, promotion of anergy by diversion to regulatory T (Treg) cells, and a putative influence over thymic cellularity.

### Activation of PGE

Actually, PGE is not restricted to mTECs, but ts-ag-encoding genes expressed in cTECs are mostly lymphocyte-specific and are due to contamination by thymocytes complexed to thymic nurse cells, while those expressed in DCs and macrophages are mostly related to bone marrow-derived cell lineages (14). Recent studies indicate that Aire modulates, by induction of chemokine signals, cTEC gene transcription, and at the same time slows down cTEC metabolism and differentiation (204, 205). By contrast, Aire initiates PGE in mTECs (14, 202), as confirmed in fetal thymic organ cultures and cultures from adult thymi (206, 207). The process involves hundreds of genes whose expression overrides the ordinary sex-, tissue-, and differentiation-dependent regulation (14, 202, 208). However, Aire activates the transcription of a part of these genes, as demonstrated in *Aire*^−/−^ mice (209, 210). Moreover, Aire-dependent and Aire-independent genes participating in PGE co-localize in chromosomal clusters (208–210): as seen, this phenomenon is due to the localization of Aire-containing multimolecular complexes in chromatin stretches enclosing the transcription start sites of the ts-ag-encoding genes (177).

Interesting data are available when, taking into consideration a set of functionally connected genes, thymic PGE is compared with the corresponding expression in the relevant peripheral tissue. For example, while murine casein genes (clustered on chromosome 5) are co-expressed in about 90% of mammary-gland cells of young female mice, the expression of the same genes in CD80^hi^ mTECs exhibits a prevalence between 2 and 15%. The rate of mRNA translation into the respective proteins is even lower, so that each ts-ag is traceable in about 1–3% of mTECs (211). With regard to allele pairs, many mTECs use one chromosomal locus, with no obvious imprinting (212). At the same time, genes ordinarily imprinted in the peripheral tissues, such as the Aire-dependent gene encoding the insulin-like growth factor 2, may be expressed biallelically in mTECs (209). Another proof that gene transcription activated by Aire is regulated differently from the peripheral tissues is given by the observation that a selective deficiency in the pancreatic-duodenal homeobox 1, a master transcription factor encoded by an Aire-dependent gene, does not impair the thymic transcription of other Aire-dependent, pancreatic-islet-related genes (213).

Initially, although it was observed that clustered ts-ag-encoding genes have a higher chance of sharing the same fate, no clear pattern of co-expression emerged (211, 212). Recent studies have changed this perspective. Actually, single human mTECs shift through distinct pools of ts-ag-encoding genes. In this sense, some co-expression pools of overlapping and complementary gene sets have been individuated, which encompass intra- and inter-chromosomal distribution and align along a co-linear program of differentiation (214). Analogously, clustered Aire-dependent genes are expressed stochastically in small groups of murine mTECs^hi^, with a significant degree of diversity between individuals (215, 216).

Other important observations may be added: first, Aire favors alternative mRNA splicing, which represents a broadening of thymic self-representation (184–186). Second, the pool of genes regulated by Aire is conditioned by the cellular environment, as demonstrated both in physiologic conditions, by comparing mTECs with the germ cells of the testis, where PGE addresses pulsed waves of scheduled apoptosis (217), and in experimental setting, by transfecting pancreatic-islet β-cells with *Aire* (218). Finally, the dichotomy between Aire-dependent and Aire-independent genes represents perhaps an improper simplification: it is possible that genes belonging to both categories are connected into transcriptional networks that recognize a hierarchy. This could explain how Aire regulates indirectly some genes, albeit an interaction with other transcription factors cannot be excluded (219, 220).

### Self-ag Presentation and Transfer

Kuroda et al. found that *Aire*^−/−^ mice display Sjögren’s syndrome-like disease of the exocrine glands, and this was associated with autoimmunity to the ubiquitous protein α-fodrin. Surprisingly, the expression of the encoding gene was not impaired by Aire deficiency, and the authors hypothesized that the autoimmune process was due to suboptimal antigen presentation and transfer (221). Initially, the features and timing of self-ag presentation by mTECs and thymic DCs were evaluated without taking into account Aire role (222, 223). Later, Hubert et al. found that some self-ags need to be transferred to the thymic DCs to be presented to the thymocytes, and that Aire is able to address this interplay (224). In another study, lethally irradiated mice transplanted with bone marrow deficient in the gene encoding the MHCII-transactivator—hereby forced to use only antigen-presenting cells (APCs) of epithelial lineage—had a higher frequency of T-cell clones with self-reactivity to an epitope of the interphotoreceptor retinoid-binding protein (Irbp), which is encoded by an Aire-dependent gene (225). More recently, it has been demonstrated that Aire^+^ mTECs release vesicles of endocytic origin called exosomes, which carry a high number of self-ags (226).

By contrast, another research group has provided evidence that mTECs^hi^, through the process of macroautophagy, induce autonomously a proper thymocyte response (227). Interestingly, both Aire^+^ mTECs and DCs, when co-cultured with fresh thymocytes, act as APCs and re-propose *in vitro* the process of negative selection (228, 229).

At this point, it seems to be correct to state that self-ag presentation by mTECs and thymic DCs runs in parallel, but preeminence and degree of redundancy of the two sources remain to be deciphered (230). In a very recent study, Mouri et al. employing transgenic mice in which ovalbumin expression has been driven by either *Aire* or rat *Ins* promoter, delineate a division of labor between mTECs and thymic DCs, which configures uneven dependency on Aire and different outcomes in central tolerance (that is, negative selection versus Treg-cell generation) (231). To complicate matters, other recent data suggest that even thymic B cells display *Aire* expression and participate in self-ag presentation (232).

### Generation of Treg Cells

**Figure 5:**
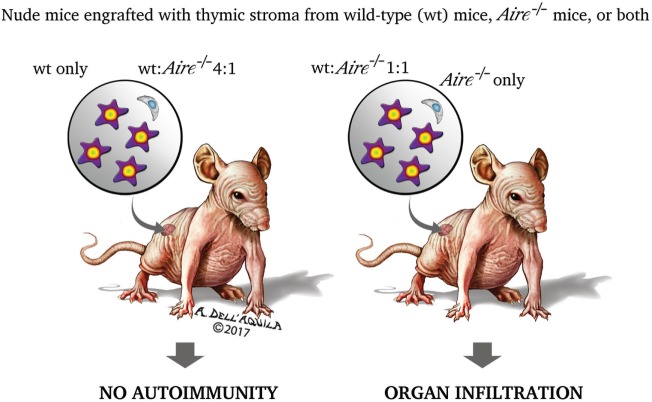
Two research group found that the organ damage of nude mice co-engrafted with 2′-deoxyguanosine-resistant thymic stroma from wild-type and *Aire*^−/−^ donors matched that of the animals engrafted with a single *Aire*^−/−^ stroma (221, 233). This led the authors to speculate that impairment of regulatory T cells (Treg cells) has no role in the autoimmune process caused by Aire deficiency. If it had, Treg cells generated in the wild-type stroma would prevent the onset of the disease. However, mice co-engrafted with an excess (4:1) of wild-type stroma had no organ damage, raising reasonable doubts on the earlier conclusions. The experiment was replicated with splenocytes co-transferred into recombinase-activating gene-1-deficient (*Rag1*^−/−^) mice, and yielded similar results.

As touched upon previously, thymus role in promoting self-tolerance relies not only on the process of negative selection, but also on the generation of Treg cells able to prevent and control the autoimmune process. Treg cells have a CD4^+^CD25^+^ phenotype and require FoxP3 to differentiate: initial studies suggested that *Aire*^−/−^ mice have a normal number of circulating CD4^+^CD25^+^ cells (201–203), which, however, do not consist solely of the Treg-cell subset. Later, Anderson et al. (Figure 5) observed that nude mice co-engrafted with 2′-deoxyguanosine-resistant thymic stroma from wild-type and *Aire*^−/−^ mice, or, in an alternative approach, recombinase-activating gene-1-deficient (*Rag1*^−/−^) mice treated with co-transfer of splenocytes from the above donors, exhibit organ infiltrates undistinguishable from those found in the animals engrafted with a single *Aire*^−/−^ thymus or receiving splenocytes from *Aire*^−/−^ mice only. Should Treg-cell impairment play a role in the adverse events deriving from Aire deficiency, generation of Treg cells in the co-engrafted wild-type thymus (or their presence among the co-transferred wild-type splenocytes) would prevent the autoimmune process. However, avoiding organ damage by introduction of an excess of thymic stroma (or splenocytes) from wild-type animals left reasonable doubts on the earlier conclusions (233). Similar data were obtained by Kuroda et al., albeit in this case co-engrafted thymi were employed at a fixed ratio (221). In a further study, Aire sufficiency did not enlarge, compared with a condition of Aire deficiency, Treg-cell TCR specificities on a background of TCR oligoclonality (234), but such experimental design (i.e., the utilization of transgenic mice with a restricted TCR repertoire) was questionable in itself.

On the other hand, the hypothesis of an Aire role in generating Treg cells moves from the observation that Aire deficiency exacerbates the organ damage in *FoxP3*-deficient (*FoxP3*^−/−^) mice (235). In this sense, a series of studies prove that self-ag presentation by Aire^+^ mTECs shapes the Treg-cell repertoire (227, 236–238). Critical factors for this process, whose efficiency correlates inversely with Treg-cell differentiation, are optimal affinity/avidity in TCR engagement and proper cytokine availability (237). Other observations propose a reissue of the relationship between Aire and conventional (effector) T cells: first, Aire^+^ mTECs act autonomously as APCs (227, 238), but cooperation with thymic DCs may be required for some self-ags (239, 240). Second, in the perinatal age Aire promotes the generation of a distinct compartment of Treg cells that persists into adulthood (241).

Some studies suggest that Aire promotes Treg-cell enrichment in the secondary lymphoid organs: *Rag1*^−/−^*Aire*^+/+^ recipients of T cells from *Aire*^−/−^ mice show hyperproliferation of the FoxP3^+^ subset able to prevent overt autoimmunity (242). Coherently, consequences of Aire deficiency are made critical by constitutional defects or derailment of the mechanisms enabling Treg-cell action in the periphery (243, 244).

Unfortunately, given that Aire promotes central tolerance also to cancer-associated self-ags, generation of Treg cells with the related TCR specificities is a way to exert such unfavorable action (245–248). Finally, it is important to remember that, beside FoxP3^+^ major population, minor subsets of Treg cells exist: one of these, represented by CD8^+^CD28^lo^ T cells, fails to control the onset of experimental colitis in *Aire*^−/−^ mice (249).

### Control of Thymic Cellularity

**Figure 6:**
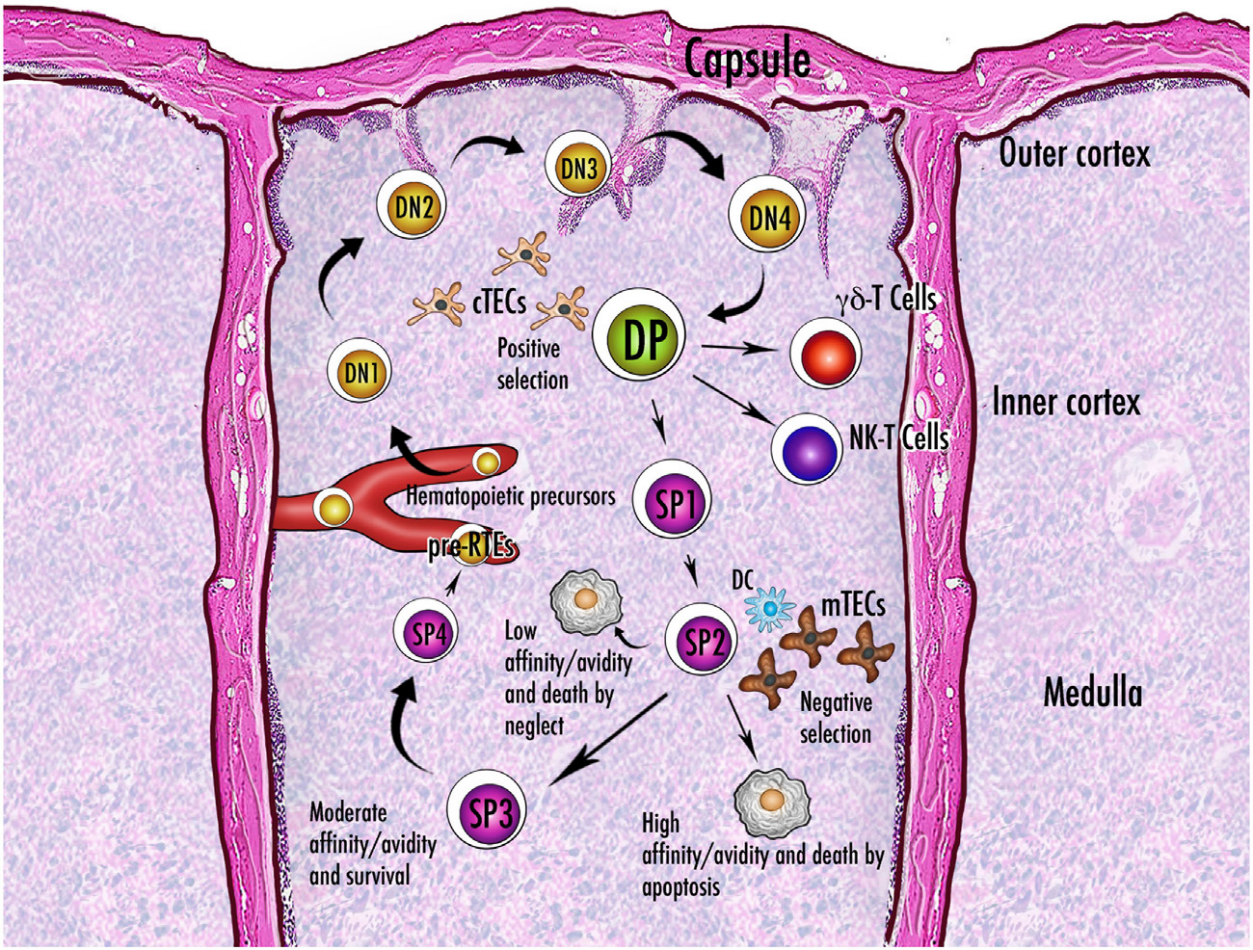
Schematic representation of thymocyte maturation and the processes of positive and negative selection. Thymocytes originate from bone marrow-derived pluripotent precursors, which in the most immature form lack the clusters of differentiation CD4 and CD8 and are referred to as double-negative (DN) cells. There are four stages (DN1–DN4) of DN condition, during which thymocytes move from the cortico-medullary junction to the subcapsular zone of the gland. The irreversible commitment to T-cell lineage intervenes in the passage from DN1 to DN2 condition, when expression of the recombinase-activating genes starts: only cells that succeed in rearrangement of the gene encoding the T-cell receptor (TCR) β-chain are selected for further maturation. After a brief DN4 stage, survived thymocytes become double-positive (DP) by acquisition of CD4 and CD8 and are allowed to rearrange the gene encoding the TCR α-chain. Now they deal with the processes of positive and negative selection and their fate is dictated by the interaction with the thymic stroma. Once positively selected to generate single-positive (SP) cells, the thymocytes reverse their direction and enter the medulla. While a large percentage of thymocytes do not run into an antigen fitting their TCR and die by neglect, the fate of the remainders depends on the degree of affinity with the cognate antigen. Generally, a strong affinity induces apoptosis and ensuing clonal deletion. An intermediate affinity may induce diversion to an anergic state, such as that of the regulatory T cells. In the theory of avidity, the amount of antigen determines the outcome of the interaction. There are three or four stages of maturation of the SP thymocytes, which finally reach the perivascular space as pre-recent thymic emigrants (pre-RTEs). Abbreviations: NK, natural killer; cTECs, mTECs, thymic epithelial cells (cortical, medullary); DC, dendritic cell.

A putative role attributed to Aire is that of controlling mTEC molecular mediators that regulate thymic cellularity and dynamics (Figure 6). In this context, mTECs do not act as APCs, and their non-TCR-mediated influence relies on the production and release of cytokines.

The most important event is the cortex-to-medulla migration of the positively selected αβ-thymocytes. This non-inertial movement is elicited by various CC-chemokine ligands (CCLs) through their respective CCRs: CCL5, CCL17, and CCL22 interact with CCR4, while CCL19 and CCL21 interact with CCR7 (250). A lack of cytokine signal does not prevent thymocyte accumulation in the cortex and outflow from the thymus, but the process of negative selection is compromised (251). Conversely, after relocation, surviving single-positive thymocytes complete their maturation in three or four stages and enter the bloodstream as “recent thymic emigrants” (RTEs) (252). As reviewed by Cowan et al., intrathymic thymocyte migration is indispensable for the emergence of Treg-cell precursors, and involves at the same time thymocyte sublineages deputed to innate immunity, such as γδ-thymocytes and invariant natural-killer (iNK)-T cells (253).

Aire intervention in these events remains unclear. Laan et al. found that, in the murine thymus, Aire deficiency impairs the expression of the genes encoding CCR4 and CCR7 ligands, albeit the cellular source of the latter would not coincide with Aire^+^ mTECs (254). Other research groups found that LtβR signaling directs chemokine release by mTECs (81, 82, 85). Later, Lkhagvasuren et al. clarified that CCL21^+^ mTECs represent a distinct, LtβR-driven mTEC subset that emerges after the perinatal period and mostly segregates from Aire^+^ elements (93). A Chinese research group has dedicated a series of studies to the intra-medullary maturation of the CD4^+^ thymocytes, highlighting that a perinatal reduced outflow of RTEs, which play an important role in the establishment and maintenance of peripheral tolerance, deteriorates the detrimental effects of Aire deficiency (255–258). Further studies have been dedicated, with not univocal results, to Aire role in the generation, intrathymic migration and maturation of γδ-thymocytes, iNK-T cells, and DCs (259–263). Interestingly, recent studies suggest that Aire intervenes in regulating generation and function of Il17-releasing invariant and adaptive T cells, which have been linked to the early stage of the autoimmune processes (264, 265).

## Organ Targeting in AIRE Deficiency

Experimentally, thymic deletion of a ts-ag-encoding gene leads ineluctably to the onset of the related autoimmune disease. Given that two murine *Ins* genes (*Ins1* and *Ins2*) exist, only the latter being Aire-dependent (14, 202), Fan et al. used Cre-Lox recombination technology to restrict *Ins2* deletion to *Aire*-expressing cells. Therefore, diabetes developed within 3 weeks. Murine strain was autoimmune-resistant and the animals displayed unimpaired tolerance to other self-ags. Importantly, the authors employed *Ins1*^−/−^ mice to eliminate the interference of an Aire-independent factor, whose strength in the mechanisms of self-tolerance is undetermined (266). In the preceding study of DeVoss et al., nude mice engrafted with thymic stroma in which the *Irbp* gene was deleted, exhibited eye disease (267).

In contrast, *Aire*^−/−^ condition causes a dysregulation, mostly a downgrade of expression, of the entire pool of Aire-dependent genes. In this chaotic perturbation of thymic PGE, the pathological consequences are determined by factors acting at various levels.

### Species Specificity and Genetic Background

First, species-specific peculiarities cause remarkable differences between human APS1 and the phenotype of *Aire*^−/−^ mice: in other words, the animal models of disease exhibit pathological findings not comparable with those of the APS1 patients (268–270). Nevertheless, studies on *Aire*^−/−^ mice have made it possible to identify, with proven or potential connection to the human field, several targets of autoimmunity (271–278).

Moving to the intra-species level, the genetic background, more than *Aire* genotype, influences severity of disease and set of organs damaged in each individual, albeit in APS1 patients this is observable with some difficulty. To give a few examples of the link between geo-ethnic patient origin and clinical picture, Finnish APS1 patients have an increased prevalence of T1D (131), while autoimmune thyroiditis is common among those from Southern Italy (279). Again, chronic candidiasis is observed rarely in Iranian-Jewish APS1 patients, who generally exhibit a milder phenotype (280). It is not surprising that MHCII alleles are relevant to these differences (281).

Of course, the availability of highly inbred animal lines gives greater visibility to the phenomenon: murine autoimmune-prone strains, such as non-obese diabetic (NOD) and SJL mice, show a consistent and specific pattern of organ infiltration and self-reactivity. A relatively autoimmune-resistant strain, BALB mouse, has an intermediate prevalence of organ damage, which preferentially involves stomach and genital apparatus. Finally, an autoimmune-resistant strain, C57BL/6 (B6) mouse, shows a few components of the disease, with elective targeting to retina and prostate (221, 282). Susceptible alleles of the modifier loci—once again with privileged reference to MHCII ones—are necessary, but not always sufficient, to elicit organ damage: for example, the H2-Aβ^g7^ allele was required to induce autoimmune pancreatitis in NOD *Aire*^−/−^ mice, but was not sufficient when transferred to a B6 background (282). A related, unexpected phenomenon is the intra-organ targeting switch: typically, in NOD *Aire*^−/−^ mice the autoimmune pancreatitis hits the exocrine part of the gland, and the release of autoantibodies to an acinar-cell self-ag complements the process (283).

Studies on murine chimeras add interesting data: engrafting 2′-deoxyguanosine-resistant thymic stroma from BALB or B6 *Aire*^−/−^ mice into mirror nude animals, Han observed that Aire deficiency simply enhanced the restricted predisposition to autoimmunity of the recipients, independently from the genetic background of the donors. By contrast, when thymic stroma from NOD *Aire*^−/−^ mice was engrafted, it dictated the spectrum of organ damage, indicating that Aire deficiency impinges on a constitutional derailment of PGE (284).

### Gene Expression Variability

Not only the genetic background with which Aire deficiency overlaps, but also factors intrinsically related to *Aire* expression should be taken into account. Various studies demonstrate that the amount of mRNAs transcribed from murine Aire-dependent genes correlates with the level and timing of *Aire* mRNA (285–287), even in single cells (190). Age is an important factor able to modulate *Aire* expression: MHCII^+^ mTECs increase dramatically after birth and peak at 4 weeks of age (57). It is probable that the perinatal lymphopenia and ensuing lymphopenia-induced proliferation of *Aire*^−/−^ mice are related to the infringement of the above trend and contribute to their pathological findings (288), which are reminiscent of the 3-day-thymectomized mice described by Miller (289). At the opposite, thymic involution, as depicted in 12-month-old mice, is featured by a fall in mTEC/cTEC and MHCII^hi^/MHCII^lo^ ratios (57), and is caused by programmed aging of the primary lymphoid organs (290). The efficiency of the process of negative selection in the embryonic and neonatal thymus is confirmed by the study of Guerau-de-Arellano et al., who used a doxycycline-regulated transgene to control *Aire* expression, and found that self-tolerance established in the perinatal age is longstanding. The autoimmunity triggered by Aire deficiency was attenuated by transfer of previously tolerized T cells. Not surprisingly, lethal irradiation during *Aire* turn-off recreated the disease in adult mice (291). As cited earlier, a recent study highlights that Aire influences also the perinatal generation of Treg cells (241).

Sexual hormones seem to modulate central tolerance, explaining gender differences in susceptibility to autoimmunity. While castration prevents the decrease in thymic PGE observed in adult mice of either sex (292), androgens enhance *Aire* transcription and estrogens induce opposite changes acting at an epigenetic level (293, 294).

These physiologic variables score life periods at species level, but what can we say about inter-individual differences? Reappraising previous studies (11–13), Taubert et al. reiterated that human mTECs present a strong inter-individual disparity in *AIRE* expression and PGE. However, while mRNA from AIRE-independent genes displays restricted fluctuations uncorrelated with *AIRE* mRNA, variability in the transcription of the AIRE-dependent genes is wider and follows an obvious *AIRE*-related trend (295). Given that Liston et al., employing mice in which one *Aire* allele was deleted, found that PGE affects in quantitative terms the magnitude of self-reactive T cells escaping negative selection (296), it has been hypothesized that conditions of partial AIRE deficiency may represent a risk for non-syndromic autoimmunity when acting in synergy with other susceptibility factors. However, various research groups did not find an increased prevalence of *AIRE* variants among patients with sporadic, especially endocrine, autoimmune diseases (297–303). Instead, various patient reports suggest that some *AIRE* variants encode mutated chains that co-localize with the wild-type protein and undermine the activity of the oligomeric structure in a dominant manner (304–307). Reporter gene assays, *in vitro* structure modeling and homologous murine constructs validate such hypothesis (305–309). The resulting clinical picture is characterized by late-onset autoimmunity, milder phenotype than APS1, and incomplete penetrance (304–307).

The animal models of disease add valuable data that, once again, stress the importance of the genetic background: mTECs of murine autoimmune-prone strains display lower amounts of mRNAs from *Aire* and selected ts-ag-encoding genes (207, 310), and such dysregulation becomes more obvious in the stages preceding the overt disease (311). Based on the study of Venanzi et al., the difference relies on the strength of responsiveness to Aire and is no longer apparent when *Aire*^−/−^ strains are compared. In the same study, the authors demonstrated that, similarly to the human thymus, there are marked inter-individual differences in the thymic expression of most ts-ag-encoding genes, even between mice homogeneously fed and housed: once again, the coefficient of variation is higher for the Aire-dependent genes and drops when the residual expression is assayed in *Aire*^−/−^ thymi (312). According to the authors’ comment, this diversity may be beneficial in preventing uniform holes in central tolerance, but at the price of an unpredictable individual predisposition to autoimmunity.

### AIRE and T1D: Special Case or Paradigm?

In author opinion, the relationship between APS1 and T1D resumes most principles regarding AIRE function. Insulin is a self-ag commonly targeted in T1D and encoded by an AIRE-dependent gene: this dependence was inferred from gene expression pattern in murine (14, 202) and human mTECs (208). Later, two research groups observed that, although class-III *VNTR* alleles induce a higher level of *INS* expression compared to class-I alleles, the thymic amount of insulin varies widely among individuals carrying the same *VNTR* haplotype and correlates better with *AIRE* expression (295, 313). Another research group demonstrated that AIRE is able to bind to class-I and class-III *VNTRs*, and that the complexes modulate *INS* expression (314).

At this point, one would expect that AIRE deficiency lead invariably to overt pancreatic-islet β-cell autoimmunity. Actually, T1D affects a minority of APS1 patients (131, 132, 315), so that it can be assumed that one or more additional factors modulate the related risk. Although initially no or weak influence was attributed to MHCII alleles (316, 317), following studies modified this perspective: Gylling et al. found that DQB1*0602 plays a protective role in the development of APS1-associated T1D (318). Similarly, Halonen et al. showed a negative correlation with DRB1*15-DQB1*0602 (281). Two later studies on APS1 patients drew once again attention to the risk conferred by the T1D susceptibility locus 2, but both research groups genotyped MHCII in a limited percentage of the sample and omitted to include these data in multivariate statistics (319, 320).

Therefore, we can conclude that AIRE exerts a chief role in the hierarchical regulation of thymic *INS* expression, but, in a condition of AIRE deficiency, unfavorable classes of *VNTR* alleles are needed to reduce *INS* transcription below a critical threshold and determine a failure in the process of negative selection. Other genetic variables, such as MHCII haplotype, may further stratify the risk by shaping organ susceptibility to autoimmunity.

## AIRE: Not only “Central”

It is known that ts-ag-encoding genes are expressed also in the secondary lymphoid organs. Nonetheless, cell lineages holding this property and the related Aire role remain unresolved issues. According to two studies, main source of extra-thymic PGE would be Aire^+^ epithelial cells located within the lymph-nodal and splenic stroma. In an experimental setting, these cells were fostered to express an ovalbumin transgene driven by the promoter of the Aire-dependent gene encoding the intestinal fatty-acid-binding protein (321). In the other setting, *Aire* promoter itself drove the expression of the gene encoding the pancreatic-islet β-cell-specific glucose-6-phosphatase-related protein, a self-ag routinely undetectable in the thymus (322). Both cell types induced deletion of the TCR-specific CD8^+^ T-cell clones, even when the latter were transferred into lethally irradiated mice transplanted with bone marrow from β2-microglobulin-deficient donors. In this way, reconstituting DCs were unable to act as APCs (321, 322). Later, one of these research groups revised the phenotype of the extra-thymic Aire^+^ cells, identifying them in unconventional CD45^lo^Epcam^+^MHCII^hi^CD80^lo^ bone marrow-derived APCs (323).

Reappraising their previous study (266), Grupillo et al. deleted *Ins2* in *CD11c*-expressing cells of *Ins1*^−/−^ mice. The splenic source of *Aire* expression and PGE was attributed to CD11c^int^MHCII^+^B220^+^ plasmacytoid DCs. Treg-cell number was unaffected, so that self-tolerance was necessarily deletional. An interesting aspect of this study was that only B6 mice crossed to adopt MHCII alleles typical of NOD mouse exhibited some degree of pancreatic-islet β-cell damage (324). Jointly, the studies of this research group (266, 324) suggest that thymic PGE plays a chief role in self-tolerance, and that thymic gene deletion causes inevitably the related autoimmune disease. By contrast, the same event in the secondary lymphoid organs leads to adverse consequences only on an autoimmune-prone genetic background.

Other researchers did not find a relationship between Aire and extra-thymic PGE (325, 326). In further studies, Aire was localized in uncharacterized cells of the secondary lymphoid organs (327), or even in the stroma of non-lymphoid organs where immune tolerance is strictly needed, such as the decidua basalis at the embryo implantation site (328).

## Another Theory of Action

The mTEC developmental theory (329), which configures an alternative hypothesis on Aire function, moves from the observation that the pharyngeal arches can generate many types of tissue, and that distinct foci of mTECs are arranged in organoids resembling typical epithelial formations. Such organization suggests that the thymic medulla forms some sort of mosaic, whose pieces follow different programs of differentiation, and that mTECs with the largest PGE are intermediate elements that progressively restrict the pool of ts-ag-encoding genes expressed (330–334). Other evidences have been called into question, such as the small percentage of mTECs in which each ts-ag is detectable (335); the dependency on Aire of differentiation-associated genes and genes encoding master transcription factors (21–23, 336, 337); and the ultrastructure of *Aire*^−/−^ thymus, in which expansion of the K8^+^ subset indicates mTEC inability to differentiate into distinct epithelial lineages (338, 339). Finally, the detection of a post-Aire mTEC stage (62) and the lack, once again in the murine *Aire*^−/−^ thymus, of hyalinized structures equivalent to the human Hassall’s corpuscles (340), would provide the conclusive proof that Aire controls mTEC differentiation, and that its scheduled disappearance is a condition for the proper implementation of the latter (341–343). As discussed above, the same arguments have been used to build and support the well-defined theory that places Aire onto the high point of mTEC differentiation.

Is *Aire* expression the end-stage of an invariant differentiation program, albeit with a stochastic pattern of PGE, or does it enable, before to be lost, multiple and predetermined programs of mTEC differentiation (344, 345)? There is still no definite answer to this question, but, in author opinion, Aire mandate remains unchanged: to accomplish the largest PGE for self-tolerance induction.

## Perspectives and Conclusion

To sum up, Aire activates the transcription of a large pool of ts-ag-encoding genes in mTECs. In Aire deficiency, missed self-ag presentation to the thymocytes determines a failure in the process of negative selection and the subsequent spreading of self-reactive T cells. The absolute heritable profile of the human related disease, APS1, suggests exciting implications on the topic of gene therapies.

A first approach is aimed at obtaining a functional thymus from ESCs. Following two studies in which murine ESCs were induced to generate TEPCs able to self-renew and foster thymocyte maturation (346, 347), two research groups replicated such results with human ESCs (348, 349). In one of these studies, TEPC re-aggregation with embryonic fibroblasts and following engraftment into nude mice led to mTEC differentiation and *AIRE* expression, albeit T-cell outflow from the thymus was short-lived (349). Similar results were achieved with human and murine induced pluripotent stem cells (350, 351).

*Aire* expression can be manipulated by immunologic (78, 79, 240), virus-based (285, 352), physical (286, 287), and chemical (353) methods. Nonetheless, enhancing *Aire* expression may impair unexpected forms of immune defense and get unwelcome surprises. As seen, genes encoding some cancer-associated ts-ags are Aire-dependent (245–248), so that it is not surprising that *Aire*^−/−^ mice are able to provide a stronger immune response after melanoma challenge (354, 355).

Translating these data to hypothetical therapies of human autoimmune diseases, the cited studies suggest that, while restoring *AIRE* expression is the goal of gene therapy in APS1 patients, ideal profile of a tailored, AIRE-based treatment should be restricted to selected cell lineages or single AIRE-dependent genes, to avoid the pitfalls of a generalized PGE distortion.

Of course, just an increasing knowledge of PGE and the related Aire role will help to refine any strategy aiming at restoring, promoting, or strengthening the mechanisms of central and peripheral self-tolerance. Finally, author refers the kind readers to excellent preceding reviews, which recapitulate the course of discoveries over Aire, and mark chronologically doubts and insights into its function (356–380).

## Author Contributions

RP is the only contributor to the Review.

## Conflict of Interest Statement

The author declares that the research was conducted in the absence of any commercial or financial relationships that could be construed as a potential conflict of interest.

## Abbreviations

Ac, acetylation; AIRE/Aire, autoimmune regulator; APC, antigen-presenting cell; APS1, autoimmune polyglandular syndrome type 1; ATF7IP, activating-transcription-factor-7-interacting protein; Brd4, bromodomain-containing domain 4; CARD, caspase-activation and recruitment domain; CBP, CREB-binding protein; CCL, CC-chemokine ligand; CCR, CC-chemokine receptor; DC, dendritic cell; DNA-PK, DNA-activated protein kinase; DNA-TOP/DNA-Top, DNA topoisomerase; E, embryonic day; EpCAM, epithelial-cell adhesion molecule; ESC, embryonic stem cell; Fgf, fibroblast growth factor; Fox, forkhead-box; HIPK2/Hipk2, homeodomain-interacting protein kinase 2; Il, interleukin; iNK, invariant natural killer; *INS*/*Ins*, insulin gene; Irbp, interphotoreceptor retinoid-binding protein; K, keratin; LtβR, lymphotoxin-β receptor; MBD1, methyl-CpG-binding-domain protein 1; MHC, major histocompatibility complex; NB, nuclear body; NLS, nuclear localization signal; NOD, non-obese diabetic; PARP1, poly-(ADP-ribose) polymerase 1; PGE, promiscuous gene expression; PHD, plant-homeodomain; P-TEFb/P-Tefb, positive transcription elongation factor b; *Rag*, recombinase-activating gene; Rank, receptor activator of nuclear factor Nf-κB; RTE, recent thymic emigrant; self-ag, self-antigen; Sf, super-family; T1D, type-1 diabetes; TCR, T-cell receptor; TEC (cTEC, mTEC), thymic epithelial cell (cortical, medullary); TEPC, thymic epithelial progenitor cell; Tnf, tumor necrosis factor; TnfR, tumor necrosis factor-receptor; Treg cell, regulatory T cell; ts-ag, tissue-specific antigen; UEA1, *Ulex europaeus* agglutinin 1; *VNTR*, variable number of tandem repeat.
