# Supplementary material for: Twenty Years of AIRE
Source: Front Immunol. 2018 Feb 12;9:98. doi: 10.3389/fimmu.2018.00098 (PMC5816566; doi:10.3389/fimmu.2018.00098)
Supplement: Supplementary file 1 [file Table_1.PDF]

Table S1. Summary of functions attributed to thymic crosstalk molecules and related experimental defects. Evidences coming from defects of molecules involved in TCR engagement and co-stimulation are reported as well

**Tnf-Sf/TnfR-Sf molecules**		**References**
Ltα-Ltβ-LIGHT/LtβR	This axis promotes mTEC generation by upregulating *Rank* expression in mTEC precursors. In postnatal thymus, LtβR signaling drives post-Aire mTEC differentiation	(92, 101)
	According to a research group, LtβR signaling controls directly *Aire* expression, and in mice deficient in genes encoding the related molecules Aire^{+} mTECs are severely decreased. LtβR induces release of CCR7 ligands	(78–81)
	Other studies indicate that *Ltα*$^{-/-}$*LIGHT*$^{-/-}$ and *LtβR*$^{-/-}$ conditions cause moderate disorganization of thymic medulla, made severe by co-occurring deficiency in Rank (but not CD40) signaling. mTECs are proportionally reduced, but *Aire* expression is unimpaired. Induction of CCR7-ligand release is confirmed	(82–85, 92, 93, 107)
	Most recent studies demonstrate that LtβR signaling controls thymic DC population	(107)
	Ltα and *Ltα-Ltβ* overexpression cause precocious thymic involution (attenuated on *LtβR*$^{-/-}$ background)	(99)
RankL/Rank	In embryonic thymus, RankL/Rank axis is essential for generation of mTECs. *Rank* expression is upregulated by LtβR signaling and thereafter increases by self-amplifying loop. In postnatal thymus, Rank signaling contributes to maintenance of mTEC proliferation and size	(91, 92)
	Lti cells and DETCs provide first RankL release in embryonic thymus. In adult thymus, single-positive thymocytes (above all recently positively selected CD4^{+}) and iNK-T cells relieve this function	(98, 100, 102–104, 108)
	RankL$^{-/-}$ and *Rank*$^{-/-}$ conditions cause moderate-severe disorganization of thymic medulla. mTECs^{hi} are reduced more than mTECs^{lo}	(91, 92)
	Opg is a decoy receptor for RankL exerting negative feedback in postnatal thymus. *Opg*$^{-/-}$ condition causes mTEC increase	(94, 105, 108)
CD40L/CD40	This axis has no role in embryonic thymus. In postnatal thymus, it cooperates with Rank signaling to maintain mTEC proliferation and size	(100, 103)
	CD40L is released by CD4^{+} thymocytes at a late stage of maturation	(100, 103)
	CD40L$^{-/-}$ and *CD40*$^{-/-}$ conditions cause slight disorganization of thymic medulla. Medullary defect is very severe if deficiency in Rank signaling co-occurs	(91, 92)
NIK, IκKα, RelB, Nf-κB2	These molecules are components of non-classical (alternative) Nf-κB pathway. *NIK*$^{-/-}$ (*aly*/*aly*), *IκKα* $^{-/-}$ and *RelB*$^{-/-}$ conditions cause profound disorganization of thymic medulla and almost complete absence of mTECs. Thymic	(76, 77, 86, 88–90)

	disorganization is less severe in *Nf-κB2*$^{-/-}$ mice	
	Non-classical Nf-κB pathway transduces cooperative LtβR and Rank signaling, and RelB is essential for early differentiation of mTEC precursors. In *RelB*$^{-/-}$ thymus pro-pmTECs (Ssea^{+}CD24^{hi} and Rank$^{-/lo}$) do not differentiate beyond this stage	(95, 106)
	Traf3 inhibits non-classical Nf-κB pathway. *Traf3*$^{-/-}$ condition overcomes requirement for LtβR signaling in thymic medulla	(112)
Traf6	Traf6 is a component of classical (canonical) Nf-κB pathway. Traf6 is essential for advanced differentiation of mTEC precursors. Thymic medulla is very severely disorganized in *Traf6*$^{-/-}$ mice, and mTECs are sparse	(87, 96, 97)
	If classical Nf-κB pathway is abolished, pmTECs (Ssea^{-}CD24^{lo} and Rank^{+}) retain cTEC-associated markers and do not differentiate into mTECs	(95, 106)
**APC Defects**		
MHCI, MHCII, CIITa	In combination with antigenic stimulus, MHCI and MHCII molecules are required for generation of single-positive thymocytes. CIITa activates transcription of genes encoding MHCII molecules. Deficiency in MHCII molecules (as noted in *H2-Aα*$^{-/-}$ and *CIITa*$^{-/-}$ mice) causes moderate hypotrophy of thymic medulla. mTECs^{hi} are reduced more than mTECs^{lo}	(109–111)
	K14 marks cTECs, and introduction of *K14-CIITa* transgene in *CIITa*$^{-/-}$ mice fails to restore mTEC number	(109)
CD80	CD80 marks mTECs^{hi} and binds to CD28. CD80/CD28 interaction co-stimulates thymocytes and, in cooperation with CD40L/CD40 axis, facilitates LtβR and Rank signaling. *CD80*$^{-/-}$ condition causes slight disorganization of thymic medulla. Medullary defect resembles *TCRα*$^{-/-}$ condition if deficiency in CD40 signaling co-occurs	(113)
**THYMOCYTE Defects**		
RORγ, TCRγ, TCRδ	RORγ is a nuclear receptor involved in lymphoid organogenesis. It is required for generation of Lti cells. These and DETCs (γδ-T cells) are first producers of RankL in embryonic thymus	(98, 100, 102)
	RORγ$^{-/-}$, *TCRδ*$^{-/-}$ and *RORγ*$^{-/-}$*TCRδ*$^{-/-}$ mice have normal thymic architecture, but moderate/severe reduction in Aire^{+} mTECs	(100, 102)
TCRα, TCRβ, Zap70	Single-positive thymocytes sustain formation of thymic medulla. Zap70 is a tyrosine-kinase protein deputed to mediate intracellular TCR signaling. *TCRα*$^{-/-}$ and *Zap70*$^{-/-}$ conditions (featured by absence of single-positive thymocytes) cause atrophy of thymic medulla, with small mTEC clusters	(109–111)
	Use of transgenic mice with restricted TCR repertoire shows that antigen recognition by positively selected CD4^{+}	(109, 110)

thymocytes is indispensable for maintenance of medullary size and mTEC number I

Tnf, tumor necrosis factor; TnfR, tumor necrosis factor-receptor; Sf, super-family; Lt, lymphotoxin; LIGHT, homologous to lymphotoxin, exhibits inducible expression and competes with HSV glycoprotein D for binding to herpesvirus entry mediator, a receptor expressed on T lymphocytes; LtβR, Ltβ receptor; mTEC, medullary thymic epithelial cell; pmTEC, mTEC precursor; pro-pmTEC, mTEC pro-precursor; CCR, CC-chemokine receptor; Rank, receptor activator of nuclear factor Nf-κB; RankL, Rank ligand; Lti, lymphoid tissue inducer; DETC, dendritic epidermal T cells; iNK, invariant natural killer; Opg, osteoprotegerin; NIK, Nf-κB-inducing kinase; IκKα, IκB-kinase α-subunit; Nf-κB, nuclear factor Nf-κB; Ssea, stage-specific embryonic antigen; Traf, TnfR-associated factor; APC, antigen-presenting cell; MHCI/MHCII, (class-I/class-II) major histocompatibility complex; CIITa, MHCII transactivator; K, keratin; TCR, T-cell receptor; ROR, RAR-related orphan receptor; Zap70, ζ-chain-associated 70-kDa protein
